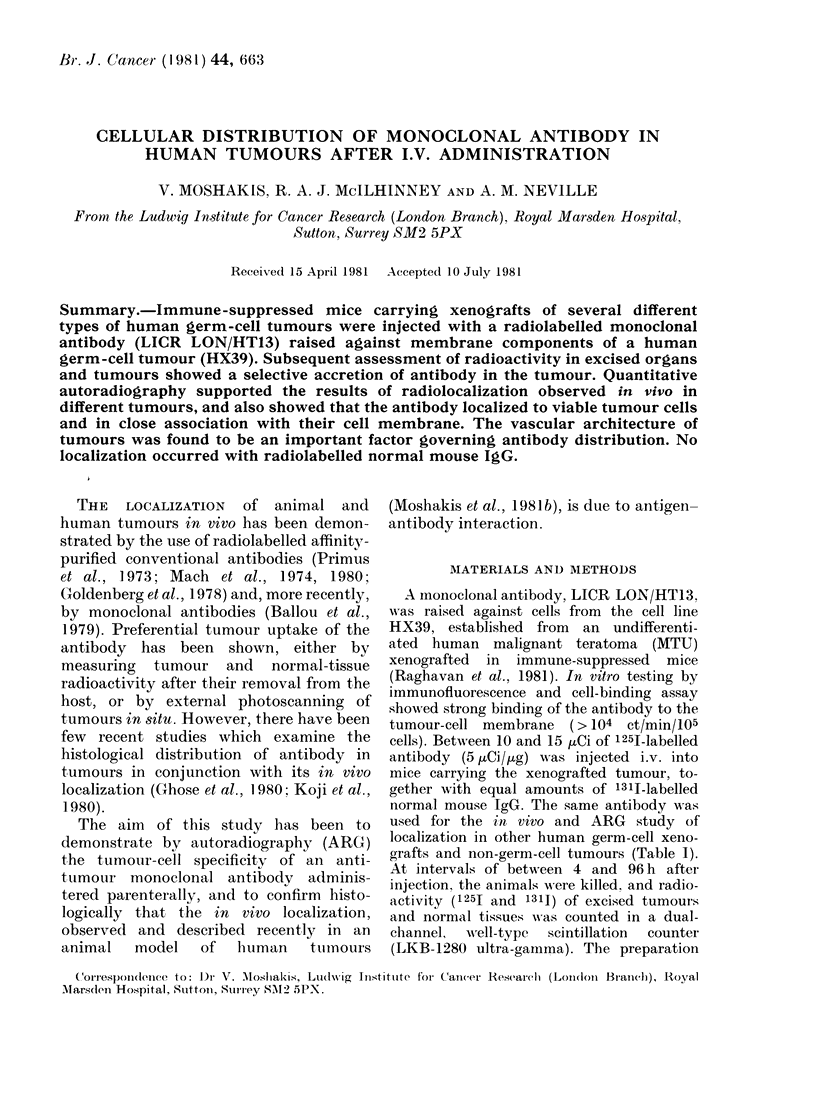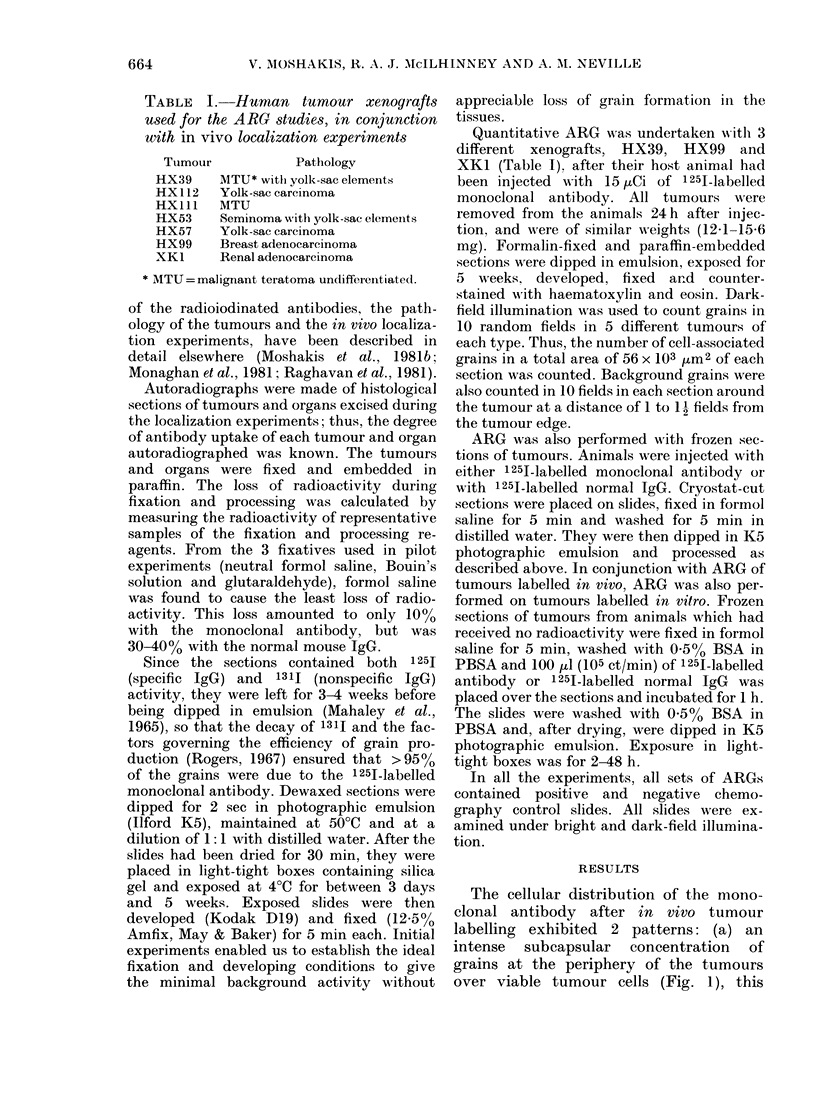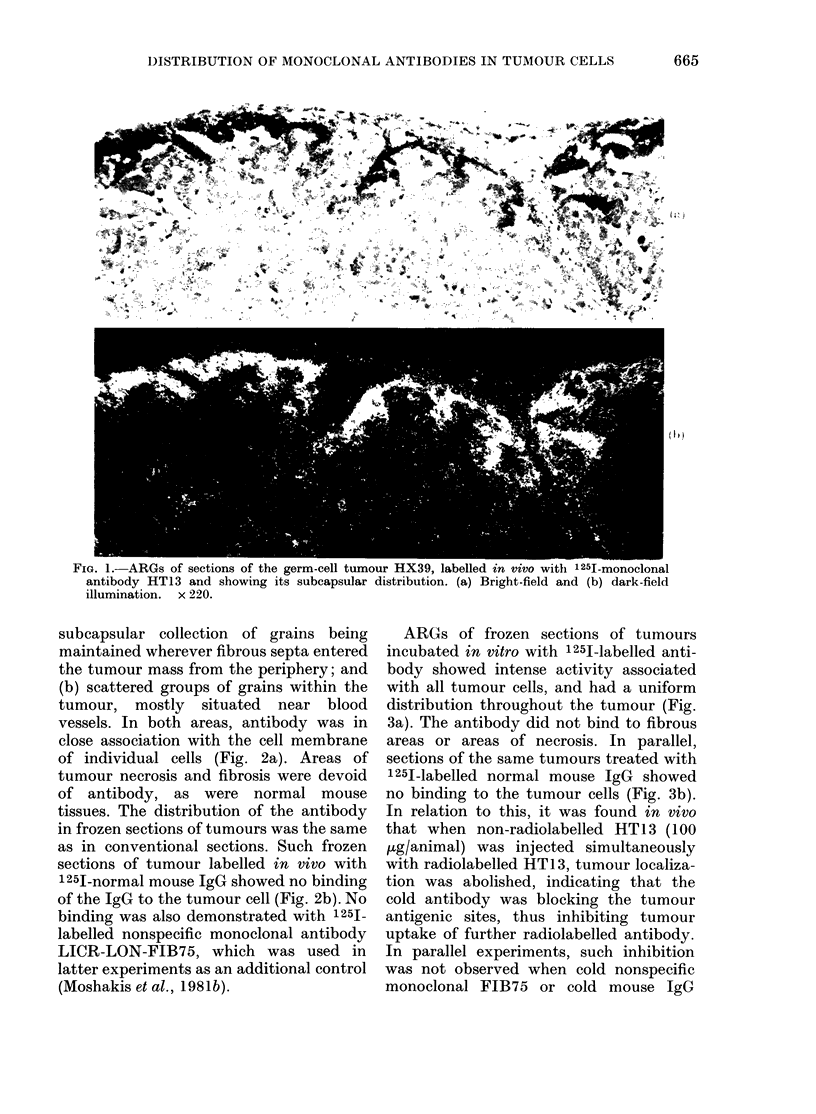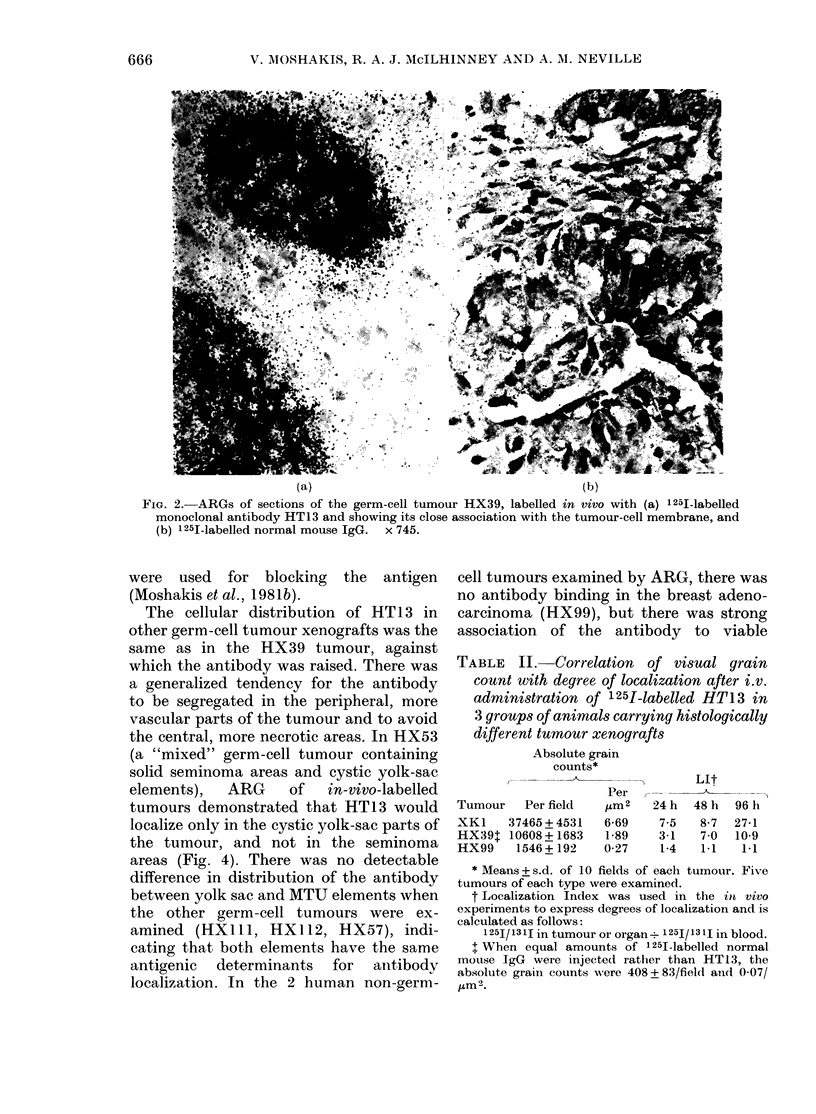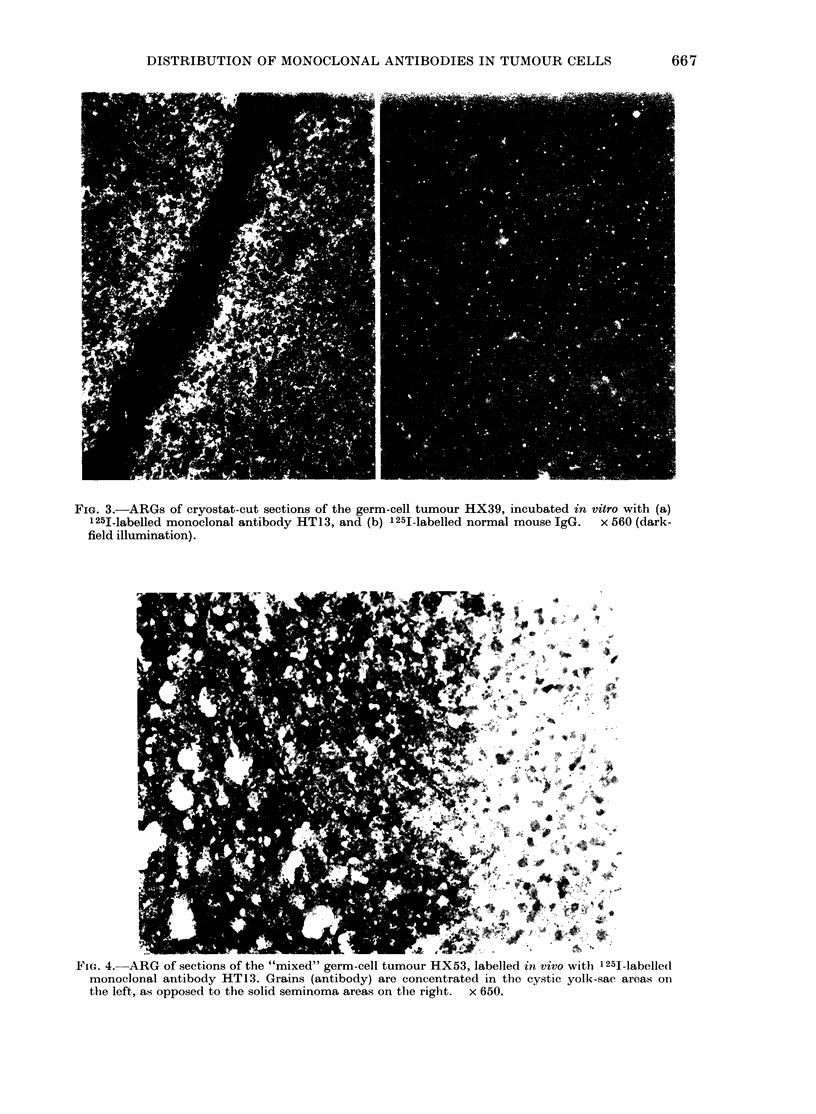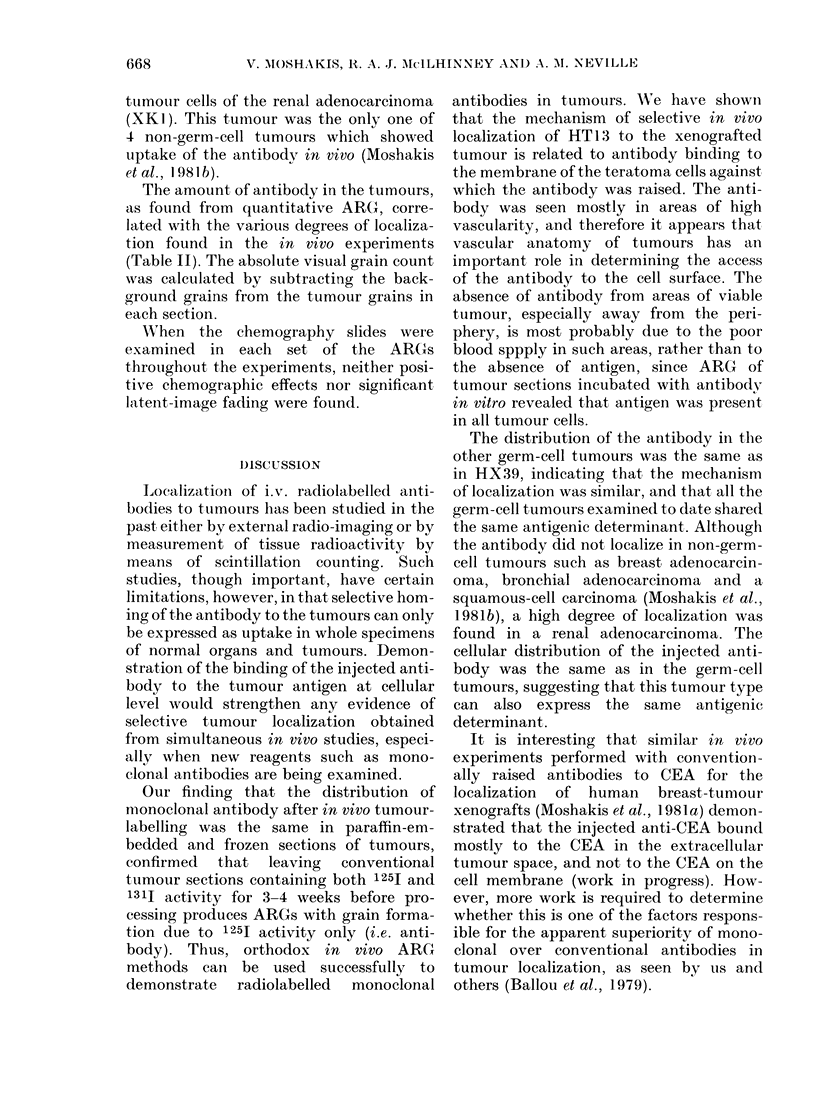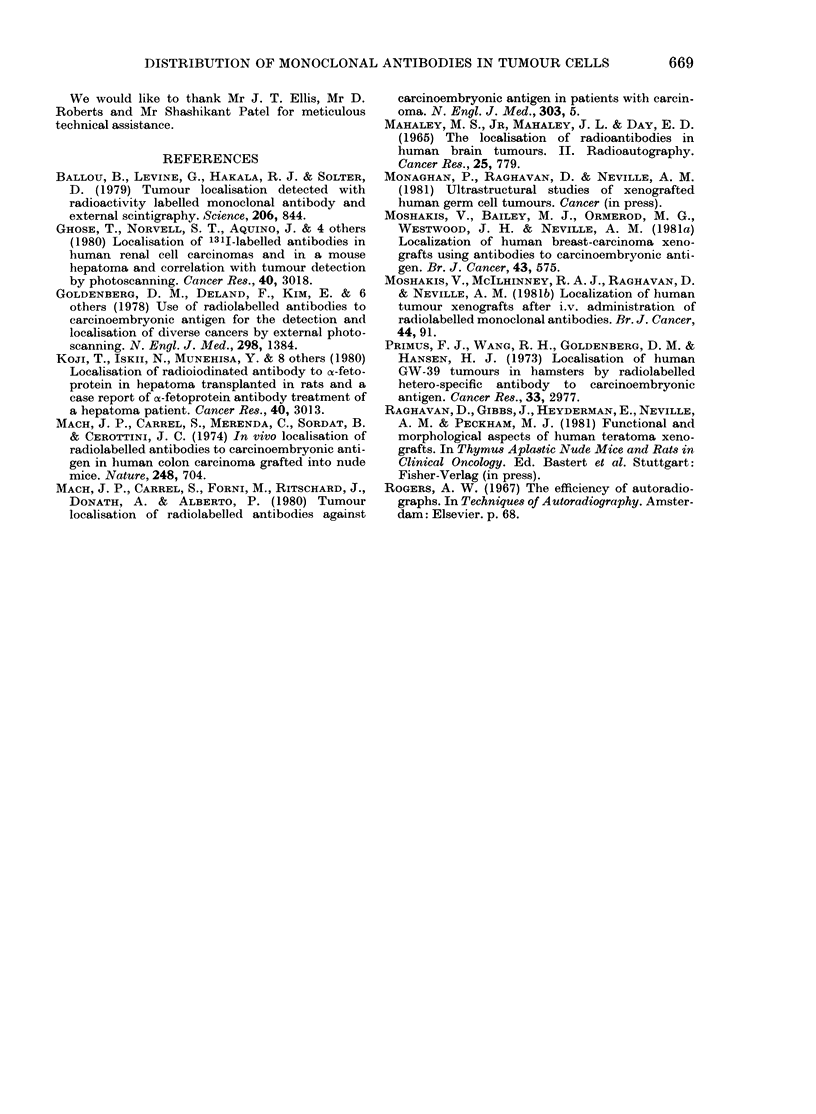# Cellular distribution of monoclonal antibody in human tumours after i.v. administration.

**DOI:** 10.1038/bjc.1981.251

**Published:** 1981-11

**Authors:** V. Moshakis, R. A. McIlhinney, A. M. Neville

## Abstract

**Images:**


*Br. J. Cancer* (1981) **44**, 663

### CELLULAR DISTRIBUTION OF MONOCLONAL ANTIBODY IN HUMAN TUMOURS AFTER I.V. ADMINISTRATION

V. MOSHAKIS, R. A. J. McILHINNEY AND A. M. NEVILLE

*From the Ludwig Institute for Cancer Research (London Branch), Royal Marsden Hospital, Sutton, Surrey SM2 5PX*

Received 15 April 1981 Accepted 10 July 1981

**Summary.—Immune-suppressed mice carrying xenografts of several different types of human germ-cell tumours were injected with a radiolabelled monoclonal antibody (LICR LON/HT13) raised against membrane components of a human germ-cell tumour (HX39). Subsequent assessment of radioactivity in excised organs and tumours showed a selective accretion of antibody in the tumour. Quantitative autoradiography supported the results of radiolocalization observed *in vivo* in different tumours, and also showed that the antibody localized to viable tumour cells and in close association with their cell membrane. The vascular architecture of tumours was found to be an important factor governing antibody distribution. No localization occurred with radiolabelled normal mouse IgG.**

THE LOCALIZATION of animal and human tumours *in vivo* has been demonstrated by the use of radiolabelled affinity-purified conventional antibodies (Primus *et al.*, 1973; Mach *et al.*, 1974, 1980; Goldenberg *et al.*, 1978) and, more recently, by monoclonal antibodies (Ballou *et al.*, 1979). Preferential tumour uptake of the antibody has been shown, either by measuring tumour and normal-tissue radioactivity after their removal from the host, or by external photoscanning of tumours *in situ*. However, there have been few recent studies which examine the histological distribution of antibody in tumours in conjunction with its *in vivo* localization (Ghose *et al.*, 1980; Koji *et al.*, 1980).

The aim of this study has been to demonstrate by autoradiography (ARG) the tumour-cell specificity of an anti-tumour monoclonal antibody administered parenterally, and to confirm histologically that the *in vivo* localization, observed and described recently in an animal model of human tumours (Moshakis *et al.*, 1981*b*), is due to antigen–antibody interaction.

### MATERIALS AND METHODS

A monoclonal antibody, LICR LON/HT13, was raised against cells from the cell line HX39, established from an undifferentiated human malignant teratoma (MTU) xenografted in immune-suppressed mice (Raghavan *et al.*, 1981). *In vitro* testing by immunofluorescence and cell-binding assay showed strong binding of the antibody to the tumour-cell membrane ($>10^4$ ct/min/$10^5$ cells). Between 10 and 15 $\mu$Ci of $^{125}$I-labelled antibody (5 $\mu$Ci/$\mu$g) was injected i.v. into mice carrying the xenografted tumour, together with equal amounts of $^{131}$I-labelled normal mouse IgG. The same antibody was used for the *in vivo* and ARG study of localization in other human germ-cell xenografts and non-germ-cell tumours (Table I). At intervals of between 4 and 96 h after injection, the animals were killed, and radioactivity ($^{125}$I and $^{131}$I) of excised tumours and normal tissues was counted in a dual-channel, well-type scintillation counter (LKB-1280 ultra-gamma). The preparation

Correspondence to: Dr V. Moshakis, Ludwig Institute for Cancer Research (London Branch), Royal Marsden Hospital, Sutton, Surrey SM2 5PX.

TABLE I.—*Human tumour xenografts used for the ARG studies, in conjunction with* in vivo *localization experiments*

| Tumour | Pathology |
|---|---|
| HX39 | MTU* with yolk-sac elements |
| HX112 | Yolk-sac carcinoma |
| HX111 | MTU |
| HX53 | Seminoma with yolk-sac elements |
| HX57 | Yolk-sac carcinoma |
| HX99 | Breast adenocarcinoma |
| XK1 | Renal adenocarcinoma |

* MTU = malignant teratoma undifferentiated.

of the radioiodinated antibodies, the pathology of the tumours and the *in vivo* localization experiments, have been described in detail elsewhere (Moshakis *et al.*, 1981*b*; Monaghan *et al.*, 1981; Raghavan *et al.*, 1981).

Autoradiographs were made of histological sections of tumours and organs excised during the localization experiments; thus, the degree of antibody uptake of each tumour and organ autoradiographed was known. The tumours and organs were fixed and embedded in paraffin. The loss of radioactivity during fixation and processing was calculated by measuring the radioactivity of representative samples of the fixation and processing reagents. From the 3 fixatives used in pilot experiments (neutral formol saline, Bouin's solution and glutaraldehyde), formol saline was found to cause the least loss of radioactivity. This loss amounted to only 10% with the monoclonal antibody, but was 30–40% with the normal mouse IgG.

Since the sections contained both $^{125}$I (specific IgG) and $^{131}$I (nonspecific IgG) activity, they were left for 3–4 weeks before being dipped in emulsion (Mahaley *et al.*, 1965), so that the decay of $^{131}$I and the factors governing the efficiency of grain production (Rogers, 1967) ensured that >95% of the grains were due to the $^{125}$I-labelled monoclonal antibody. Dewaxed sections were dipped for 2 sec in photographic emulsion (Ilford K5), maintained at 50°C and at a dilution of 1:1 with distilled water. After the slides had been dried for 30 min, they were placed in light-tight boxes containing silica gel and exposed at 4°C for between 3 days and 5 weeks. Exposed slides were then developed (Kodak D19) and fixed (12·5% Amfix, May & Baker) for 5 min each. Initial experiments enabled us to establish the ideal fixation and developing conditions to give the minimal background activity without appreciable loss of grain formation in the tissues.

Quantitative ARG was undertaken with 3 different xenografts, HX39, HX99 and XK1 (Table I), after their host animal had been injected with 15 $\mu$Ci of $^{125}$I-labelled monoclonal antibody. All tumours were removed from the animals 24 h after injection, and were of similar weights (12·1–15·6 mg). Formalin-fixed and paraffin-embedded sections were dipped in emulsion, exposed for 5 weeks, developed, fixed and counterstained with haematoxylin and eosin. Dark-field illumination was used to count grains in 10 random fields in 5 different tumours of each type. Thus, the number of cell-associated grains in a total area of $56 \times 10^3$ $\mu$m$^2$ of each section was counted. Background grains were also counted in 10 fields in each section around the tumour at a distance of 1 to 1½ fields from the tumour edge.

ARG was also performed with frozen sections of tumours. Animals were injected with either $^{125}$I-labelled monoclonal antibody or with $^{125}$I-labelled normal IgG. Cryostat-cut sections were placed on slides, fixed in formol saline for 5 min and washed for 5 min in distilled water. They were then dipped in K5 photographic emulsion and processed as described above. In conjunction with ARG of tumours labelled *in vivo*, ARG was also performed on tumours labelled *in vitro*. Frozen sections of tumours from animals which had received no radioactivity were fixed in formol saline for 5 min, washed with 0·5% BSA in PBSA and 100 $\mu$l ($10^5$ ct/min) of $^{125}$I-labelled antibody or $^{125}$I-labelled normal IgG was placed over the sections and incubated for 1 h. The slides were washed with 0·5% BSA in PBSA and, after drying, were dipped in K5 photographic emulsion. Exposure in light-tight boxes was for 2–48 h.

In all the experiments, all sets of ARGs contained positive and negative chemography control slides. All slides were examined under bright and dark-field illumination.

### RESULTS

The cellular distribution of the monoclonal antibody after *in vivo* tumour labelling exhibited 2 patterns: (a) an intense subcapsular concentration of grains at the periphery of the tumours over viable tumour cells (Fig. 1), this

FIG. 1.—ARGs of sections of the germ-cell tumour HX39, labelled *in vivo* with $^{125}$I-monoclonal antibody HT13 and showing its subcapsular distribution. (a) Bright-field and (b) dark-field illumination. ×220.

subcapsular collection of grains being maintained wherever fibrous septa entered the tumour mass from the periphery; and (b) scattered groups of grains within the tumour, mostly situated near blood vessels. In both areas, antibody was in close association with the cell membrane of individual cells (Fig. 2a). Areas of tumour necrosis and fibrosis were devoid of antibody, as were normal mouse tissues. The distribution of the antibody in frozen sections of tumours was the same as in conventional sections. Such frozen sections of tumour labelled *in vivo* with $^{125}$I-normal mouse IgG showed no binding of the IgG to the tumour cell (Fig. 2b). No binding was also demonstrated with $^{125}$I-labelled nonspecific monoclonal antibody LICR-LON-FIB75, which was used in latter experiments as an additional control (Moshakis *et al.*, 1981*b*).

ARGs of frozen sections of tumours incubated *in vitro* with $^{125}$I-labelled antibody showed intense activity associated with all tumour cells, and had a uniform distribution throughout the tumour (Fig. 3a). The antibody did not bind to fibrous areas or areas of necrosis. In parallel, sections of the same tumours treated with $^{125}$I-labelled normal mouse IgG showed no binding to the tumour cells (Fig. 3b). In relation to this, it was found *in vivo* that when non-radiolabelled HT13 (100 $\mu$g/animal) was injected simultaneously with radiolabelled HT13, tumour localization was abolished, indicating that the cold antibody was blocking the tumour antigenic sites, thus inhibiting tumour uptake of further radiolabelled antibody. In parallel experiments, such inhibition was not observed when cold nonspecific monoclonal FIB75 or cold mouse IgG

FIG. 2.—ARGs of sections of the germ-cell tumour HX39, labelled *in vivo* with (a) $^{125}$I-labelled monoclonal antibody HT13 and showing its close association with the tumour-cell membrane, and (b) $^{125}$I-labelled normal mouse IgG. ×745.

were used for blocking the antigen (Moshakis *et al.*, 1981*b*).

The cellular distribution of HT13 in other germ-cell tumour xenografts was the same as in the HX39 tumour, against which the antibody was raised. There was a generalized tendency for the antibody to be segregated in the peripheral, more vascular parts of the tumour and to avoid the central, more necrotic areas. In HX53 (a "mixed" germ-cell tumour containing solid seminoma areas and cystic yolk-sac elements), ARG of *in-vivo*-labelled tumours demonstrated that HT13 would localize only in the cystic yolk-sac parts of the tumour, and not in the seminoma areas (Fig. 4). There was no detectable difference in distribution of the antibody between yolk sac and MTU elements when the other germ-cell tumours were examined (HX111, HX112, HX57), indicating that both elements have the same antigenic determinants for antibody localization. In the 2 human non-germ-cell tumours examined by ARG, there was no antibody binding in the breast adenocarcinoma (HX99), but there was strong association of the antibody to viable

TABLE II.—*Correlation of visual grain count with degree of localization after i.v. administration of* $^{125}$*I-labelled HT*13 *in* 3 *groups of animals carrying histologically different tumour xenografts*

| | Absolute grain counts* | | LI† | | |
|---|---|---|---|---|---|
| Tumour | Per field | Per $\mu m^2$ | 24 h | 48 h | 96 h |
| XK1 | 37465 ± 4531 | 6·69 | 7·5 | 8·7 | 27·1 |
| HX39‡ | 10608 ± 1683 | 1·89 | 3·1 | 7·0 | 10·9 |
| HX99 | 1546 ± 192 | 0·27 | 1·4 | 1·1 | 1·1 |

* Means ± s.d. of 10 fields of each tumour. Five tumours of each type were examined.

† Localization Index was used in the *in vivo* experiments to express degrees of localization and is calculated as follows:

$^{125}I/^{131}I$ in tumour or organ ÷ $^{125}I/^{131}I$ in blood.

‡ When equal amounts of $^{125}$I-labelled normal mouse IgG were injected rather than HT13, the absolute grain counts were 408 ± 83/field and 0·07/$\mu m^2$.

FIG. 3.—ARGs of cryostat-cut sections of the germ-cell tumour HX39, incubated *in vitro* with (a) $^{125}$I-labelled monoclonal antibody HT13, and (b) $^{125}$I-labelled normal mouse IgG. ×560 (dark-field illumination).

FIG. 4.—ARG of sections of the "mixed" germ-cell tumour HX53, labelled *in vivo* with $^{125}$I-labelled monoclonal antibody HT13. Grains (antibody) are concentrated in the cystic yolk-sac areas on the left, as opposed to the solid seminoma areas on the right. ×650.

tumour cells of the renal adenocarcinoma (XK1). This tumour was the only one of 4 non-germ-cell tumours which showed uptake of the antibody *in vivo* (Moshakis *et al.*, 1981*b*).

The amount of antibody in the tumours, as found from quantitative ARG, correlated with the various degrees of localization found in the *in vivo* experiments (Table II). The absolute visual grain count was calculated by subtracting the background grains from the tumour grains in each section.

When the chemography slides were examined in each set of the ARGs throughout the experiments, neither positive chemographic effects nor significant latent-image fading were found.

### DISCUSSION

Localization of i.v. radiolabelled antibodies to tumours has been studied in the past either by external radio-imaging or by measurement of tissue radioactivity by means of scintillation counting. Such studies, though important, have certain limitations, however, in that selective homing of the antibody to the tumours can only be expressed as uptake in whole specimens of normal organs and tumours. Demonstration of the binding of the injected antibody to the tumour antigen at cellular level would strengthen any evidence of selective tumour localization obtained from simultaneous *in vivo* studies, especially when new reagents such as monoclonal antibodies are being examined.

Our finding that the distribution of monoclonal antibody after *in vivo* tumour-labelling was the same in paraffin-embedded and frozen sections of tumours, confirmed that leaving conventional tumour sections containing both $^{125}$I and $^{131}$I activity for 3–4 weeks before processing produces ARGs with grain formation due to $^{125}$I activity only (*i.e.* antibody). Thus, orthodox *in vivo* ARG methods can be used successfully to demonstrate radiolabelled monoclonal antibodies in tumours. We have shown that the mechanism of selective *in vivo* localization of HT13 to the xenografted tumour is related to antibody binding to the membrane of the teratoma cells against which the antibody was raised. The antibody was seen mostly in areas of high vascularity, and therefore it appears that vascular anatomy of tumours has an important role in determining the access of the antibody to the cell surface. The absence of antibody from areas of viable tumour, especially away from the periphery, is most probably due to the poor blood sppply in such areas, rather than to the absence of antigen, since ARG of tumour sections incubated with antibody *in vitro* revealed that antigen was present in all tumour cells.

The distribution of the antibody in the other germ-cell tumours was the same as in HX39, indicating that the mechanism of localization was similar, and that all the germ-cell tumours examined to date shared the same antigenic determinant. Although the antibody did not localize in non-germ-cell tumours such as breast adenocarcinoma, bronchial adenocarcinoma and a squamous-cell carcinoma (Moshakis *et al.*, 1981*b*), a high degree of localization was found in a renal adenocarcinoma. The cellular distribution of the injected antibody was the same as in the germ-cell tumours, suggesting that this tumour type can also express the same antigenic determinant.

It is interesting that similar *in vivo* experiments performed with conventionally raised antibodies to CEA for the localization of human breast-tumour xenografts (Moshakis *et al.*, 1981*a*) demonstrated that the injected anti-CEA bound mostly to the CEA in the extracellular tumour space, and not to the CEA on the cell membrane (work in progress). However, more work is required to determine whether this is one of the factors responsible for the apparent superiority of monoclonal over conventional antibodies in tumour localization, as seen by us and others (Ballou *et al.*, 1979).

We would like to thank Mr J. T. Ellis, Mr D. Roberts and Mr Shashikant Patel for meticulous technical assistance.

### REFERENCES

BALLOU, B., LEVINE, G., HAKALA, R. J. & SOLTER, D. (1979) Tumour localisation detected with radioactivity labelled monoclonal antibody and external scintigraphy. *Science*, **206**, 844.

GHOSE, T., NORVELL, S. T., AQUINO, J. & 4 others (1980) Localisation of $^{131}$I-labelled antibodies in human renal cell carcinomas and in a mouse hepatoma and correlation with tumour detection by photoscanning. *Cancer Res.*, **40**, 3018.

GOLDENBERG, D. M., DELAND, F., KIM, E. & 6 others (1978) Use of radiolabelled antibodies to carcinoembryonic antigen for the detection and localisation of diverse cancers by external photoscanning. *N. Engl. J. Med.*, **298**, 1384.

KOJI, T., ISKII, N., MUNEHISA, Y. & 8 others (1980) Localisation of radioiodinated antibody to α-fetoprotein in hepatoma transplanted in rats and a case report of α-fetoprotein antibody treatment of a hepatoma patient. *Cancer Res.*, **40**, 3013.

MACH, J. P., CARREL, S., MERENDA, C., SORDAT, B. & CEROTTINI, J. C. (1974) *In vivo* localisation of radiolabelled antibodies to carcinoembryonic antigen in human colon carcinoma grafted into nude mice. *Nature*, **248**, 704.

MACH, J. P., CARREL, S., FORNI, M., RITSCHARD, J., DONATH, A. & ALBERTO, P. (1980) Tumour localisation of radiolabelled antibodies against carcinoembryonic antigen in patients with carcinoma. *N. Engl. J. Med.*, **303**, 5.

MAHALEY, M. S., JR, MAHALEY, J. L. & DAY, E. D. (1965) The localisation of radioantibodies in human brain tumours. II. Radioautography. *Cancer Res.*, **25**, 779.

MONAGHAN, P., RAGHAVAN, D. & NEVILLE, A. M. (1981) Ultrastructural studies of xenografted human germ cell tumours. *Cancer* (in press).

MOSHAKIS, V., BAILEY, M. J., ORMEROD, M. G., WESTWOOD, J. H. & NEVILLE, A. M. (1981*a*) Localization of human breast-carcinoma xenografts using antibodies to carcinoembryonic antigen. *Br. J. Cancer*, **43**, 575.

MOSHAKIS, V., MCILHINNEY, R. A. J., RAGHAVAN, D. & NEVILLE, A. M. (1981*b*) Localization of human tumour xenografts after i.v. administration of radiolabelled monoclonal antibodies. *Br. J. Cancer*, **44**, 91.

PRIMUS, F. J., WANG, R. H., GOLDENBERG, D. M. & HANSEN, H. J. (1973) Localisation of human GW-39 tumours in hamsters by radiolabelled hetero-specific antibody to carcinoembryonic antigen. *Cancer Res.*, **33**, 2977.

RAGHAVAN, D., GIBBS, J., HEYDERMAN, E., NEVILLE, A. M. & PECKHAM, M. J. (1981) Functional and morphological aspects of human teratoma xenografts. In *Thymus Aplastic Nude Mice and Rats in Clinical Oncology*. Ed. Bastert *et al.* Stuttgart: Fisher-Verlag (in press).

ROGERS, A. W. (1967) The efficiency of autoradiographs. In *Techniques of Autoradiography*. Amsterdam: Elsevier. p. 68.